# Toxic effects of methoxychlor on the episodic prolactin secretory pattern: Possible mediated effects of nitric oxide production

**DOI:** 10.1186/1740-3391-4-3

**Published:** 2006-03-03

**Authors:** Anunciación Lafuente, Teresa Cabaleiro, Pilar Cano, Ana I Esquifino

**Affiliations:** 1 Laboratorio de Toxicología, Facultad de Ciencias, Universidad de Vigo, Campus de Orense, Las Lagunas, 32004 Orense, Spain; 2 Departamento de Bioquímica y Biología Molecular III, Facultad de Medicina, Universidad Complutense, 28040 Madrid, Spain

## Abstract

**Background:**

This work addresses the issue of whether methoxychlor (MTX) exposure may modify the ultradian secretion of prolactin through changes in the synthesis of nitric oxide (NO) induced by N^ω^-nitro-L-arginine methyl ester (L-NAME) in the hypothalamic-pituitary axis. Associated changes in dopamine (DA) content in the anterior (AH), mediobasal (MBH) and posterior hypothalamus (PH) and median eminence (ME) were evaluated.

**Methods:**

Two groups of animals (MTX and MTX+L-NAME treated) received subcutaneous (sc) injections of MTX at a dose of 25 mg/kg/day for one month. The other two groups of animals (control and L-NAME treated) received sc vehicle injections (0.5 mL/day of sesame oil), during the same period of time to be used as controls. Forty hours before the day of the experiment, animals were anaesthetized with intrapritoneal injections of 2.5% tribromoethanol in saline and atrial cannulas were implanted through the external jugular vein. Plasma was continuously extracted in Hamilton syringes coupled to a peristaltic bomb in tubes containing phosphate-gelatine buffer (to increase viscosity). The plasma was obtained by decantation and kept every 7 minutes for the measurement of plasma prolactin levels through a specific radioimmnunoassay and DA concentration by high-pressure liquid chromatography (HPLC).

**Results:**

Prolactin release in animals from all experimental groups analyzed was episodic. Mean plasma prolactin levels during the bleeding period, and the absolute pulse amplitude were increased after MTX or N^ω^-nitro-L-arginine methyl ester (L-NAME) administration. However MTX and L-NAME did not modify any other parameter studied with the exception of relative pulse amplitude in MTX treated rats. L-NAME administration to rats treated with the pesticide reduced mean plasma prolactin levels and the absolute amplitude of prolactin peaks. Peak duration, frequency and relative amplitude of prolactin peaks were not changed in the group of rats treated with MTX plus L-NAME as compared to either control or MTX treated rats. Whereas MTX decreased DA content in the ME and increased it in the AH, its content did not change in the MBH or PH, as compared to the values found in controls. Also, L-NAME administration decreased DA content in the ME as compared to controls. However, L- NAME administration to MTX exposed rats, markedly increased DA content in the ME as compared to either MTX treated or control rats. L-NAME administration increased DA content in the AH as compared to the values found in non-treated rats. However L-NAME administration to MTX exposed rats did not modify DA content as compared to either MTX treated or control rats. L-NAME administration did not modify DA content at the MBH nor in saline treated nor in MTX treated rats. However, the values of DA in the MBH in MTX plus L-NAME treated animals were statistically decreased as compared to L-NAME treated rats. In the PH, L-NAME administration increased DA content as compared to the values found in non-treated animals. L-NAME administration to MTX exposed rats also increased DA content as compared to either MTX treated or control rats.

**Conclusion:**

The results suggest the existence of an interaction between MTX and L-NAME in the modulation of the ultradian prolactin secretion at the pituitary levels. The possibility of an indirect effect mediated by changes in DA content at the ME requires further examination.

## Introduction

Methoxychlor (MTX) is a pesticide and its chemical name is 1,1,1-trichloro-2,2-bis(p-methoxy phenyl) ethane. It was first synthesized in 1893, and its commercial production in the United States began in 1946. This organochloride insecticide was found in 1.2 % of food samples in that country, representing an intake of 0.1 to 0.8 μg MTX/day for 16 to 19 year-old individuals [1].

It has been shown that MTX affects the reproductive function [2-10]. These effects were related to its estrogenic properties [11-14] although controversial data were reported [15]. The role of MTX on prolactin secretion is also controversial: although prolactin pituitary content and its in vitro release were elevated, the differences in circulating values of the hormone (single determinations), as compared to control animals, were not statistically significant [4].

Nitric oxide (NO) has been implicated in the regulation of the release of several hypothalamic molecules that are involved in pituitary hormone secretion (e.g., biogenic amines, amino acids, luteinizing hormone releasing-hormone (LHRH), and vasoactive intestinal peptide) [16-23]. NO effects on prolactin secretion are to some extent controversial. In fact, NO inhibits prolactin secretion from hemipituitary cultures [24]. Blockade of NO synthesis by the administration of N^ω^-nitro-L-arginine methyl ester (L-NAME), inhibits the preovulatory peak of prolactin [25] or attenuates the inhibitory effect of dopamine on prolactin secretion [23,26]. On the other hand, NO stimulates or inhibits prolactin secretion according with plasma estrogen levels [27]. All those studies were performed in the so-called basal conditions, although prolactin, like other pituitary hormones, is secreted following an ultradian episodic pattern [28,29].

Previous work from our laboratory has shown that basal prolactin secretion [30], as well as its ultradian secretory pattern, is changed by MTX exposure [31,32]. However, we did not determine whether MTX effects on the ultradian secretory pattern of prolactin secretion are mediated by NO production. The objective of the present study was to determine whether MTX exposure modifies the ultradian secretion of prolactin through changes in NO. Associated changes in dopamine (DA) content in the anterior (AH), mediobasal (MBH) and posterior hypothalamus (HP) and median eminence (ME) were evaluated.

## Materials and methods

### Reagents

Methoxychlor [MTX: 1,1,1-trichloro-2,2-bis(p-methoxy phenyl) ethane], N^ω^-nitro-L-arginine methyl ester (L-NAME), sodium chloride, and tribromoethanol were purchased from Sigma-Aldrich (Saint Louis, MO, USA). Heparin was purchased from LEO (Barcelona Spain).

### Animals

Adult male Sprague-Dawley rats weighing 300–320 g at the beginning of the experiment were used. They were maintained with rat chow and water available ad libitum in a room under controlled photoperiod (14 h light/10 h darkness; lights on from 07.00 to 21.00 h) and temperature (22 ± 2°C).

Four groups of 8 animals were used. Two groups of the animals (MTX and MTX+L-NAME groups) received subcutaneous (sc) injections of MTX at a dose of 25 mg/kg/day for one month. MTX was dissolved in sesame oil at a concentration of 17.5 mg/mL. The other two groups of animals (control and L-NAME treated) received sc vehicle injections (0.5 mL/day of sesame oil), during the same period of time, to be used as controls.

### Cannula implantation

Forty hours before the beginning of data collection, animals were anaesthetized with intraperitoneal injections of 2.5% tribromoethanol in saline (1 mL/100 g body weight) and atrial cannulas were implanted through the external jugular vein according to procedures used in previous studies [28,29]. This allows the animals to move freely in their cages during the bleeding period.

### Experimental design and blood sampling

On the day of data collection, conscious and freely moving rats from each group were continuously infused with 0.9 % saline (0.5 mL/h) for 4 h, beginning at 09.30 h. Animals of both control and MTX groups were intraperitoneally (ip) injected with saline 1 h before the beginning of the bleeding period. Rats of both L-NAME and MTX + L-NAME groups were ip injected with L-NAME, a NO synthase inhibitor, at a dose of 10 mg/kg in saline, 60 minutes before the beginning of the bleeding period. The doses and timing for L-NAME administration were selected according to previous data from the literature [33,23,25]. One hour after the beginning of the intravenous infusion of saline, and 15 min after the administration of 300 IU of heparin, rats were continuously bled through a peristaltic pump at a flow rate of 50 μL every 7 min. Blood samples were collected in Hamilton microliter syringes every 7 min for 3 h, from 10.30 h to 13.30 h. The samples were collected into assay tubes and were kept on ice that contained phosphate buffer (0.01 mol/L) with 0.1% gelatine to increase blood viscosity. Plasma was obtained by decantation (26 samples from each rat) after centrifugation (15 min at 3,000 rpm). Hematocrits remained stable after this bleeding protocol.

The studies were conducted in accord with the principles and procedures outlined in the NIH Guide for the Care and Use of the Laboratory Animals.

### Prolactin measurements

Prolactin levels, in all series from each rat, were determined by a specific double-antibody radioimmunoassay. The reagents were kindly supplied by the National Hormone and Pituitary Program (NHPP, Rockville, MD, USA). Prolactin values were expressed in terms of NIADD rat PRL-RP3 reference preparation. The sensitivity of the assay was 5 pg/tube. To analyze the variability of the assay, series of plasma of 10 replicates at 4 different concentrations of the prolactin standard curve were run. Coefficients of variation were 8.6, 6.4, 5.7 or 4.7 % of 1.5, 6.25, 12, 25 or 25 ng/mL in the standard curve, respectively. Samples were analyzed within the same assay to avoid inter-assay variations. This assay is used routinely in our laboratory [28,29,31,32].

### Data Analysis

To identify and characterize prolactin pulses, a computer program (Ultra-analysis), described by Van Cauter [34] and reviewed by Richard [35], was used. In this program, a pulse was defined as an increase exceeding a multiple of the dose-adjusted coefficient of variance (CV), followed by a significant decrease. The intraassay CV was calculated from values of 4 different concentrations of prolactin (at the level of 1.2, 6.25, 12 and 25 ng/mL) in its standard curve. Thus, the CV and the mean hormone level were determined for all hormone values that comprised the ascending and descending phases of each feasible pulse. The pulse was defined when this CV was triple that of the intra-assay CV determined at comparable mean prolactin levels. To test the specificity of pulse detection, a series of 26 samples from a pool of serum was analyzed using a threshold of 3 CV for prolactin pulses. Extensive simulation studies using computer-generated series indicated that, for series that have large and frequent pulses, threshold of 3 CV minimized both false positive and negative errors [36].

The pulsatile pattern of prolactin secretion was characterised by determining the mean hormone levels, the absolute and relative amplitudes of prolactin peaks, their frequency and the pulse duration. The program also calculates the mean half-life of the hormone. The absolute pulse amplitude was defined as the difference between the hormone level at the maximum of the peak and the hormone level at the preceding nadir. The relative pulse amplitude was calculated as the quotient between absolute pulse amplitude and preceding nadir value. Pulse frequency was defined as the number of pulses observed during the bleeding period. Pulse duration was the time between the beginning of the ascending phase of the peak and the end of the descending phase of the peak. The mean hormone level was calculated by the mean of all samples collected from each rat during the 3-hour period, and the average for the experimental group from the individual means. The half-life of the hormone is calculated by the tangent of the descending phase of the peak.

### Dopamine measurement

The hypothalamus and ME were quickly dissected out as described previously [46]. The AH, MBH, PH and ME were immediately dissected and homogenized in chilled (0–1°C) 2 M acetic acid. After centrifugation (at 15,000 rpm for 30 min, at 4°C), the supernatants were analyzed by high performance liquid chromatography (HPLC), using electrochemical detection (Coulochem, 5100A, ESA, USA). A C-18 reverse phase column, eluted with a mobile phase (pH = 4; 0.1 M sodium acetate, 0.1 M citric acid, 0.7 mM sodium octylsulphate and 0.57 mM EDTA containing 10% methanol, v/v), was employed. Flow rate was 1 mL/min, at a pressure of 2200 psi. Fixed potentials against H_2_/H^+ ^reference electrode were: conditioning electrode -0.4 V; pre-oxidation electrode +0.10 V; working electrode +0.35 V. Dopamine concentration was calculated from the chromatographic peak areas by using external standards. The linearity of the detector response for dopamine was tested within the concentration ranges found in supernatants of mediobasal hypothalamus [37].

### Statistics

The results of the parameters measured in this study were tested for variance homogeneity through the Snedecor test (Snedecor, 1989). When the results did not have a homogeneous variance, they were compared through a Mann-Whitney test. If the variance was homogeneous, the Student's t-test or the analysis of variance (ANOVA) was applied. The results were considered significant at P ≤ 0.05. All values represent the mean ± S.E.M. To study the MTX and L-NAME effects on the parameters that characterize the episodic prolactin secretion and on dopamine concentration, the comparison of values was done by one-way analysis of variance (ANOVA). Finally, to test the existence of an interaction between MTX and TRH, two ways analysis of variance (ANOVA) was applied.

## Results

**Figure 1:**
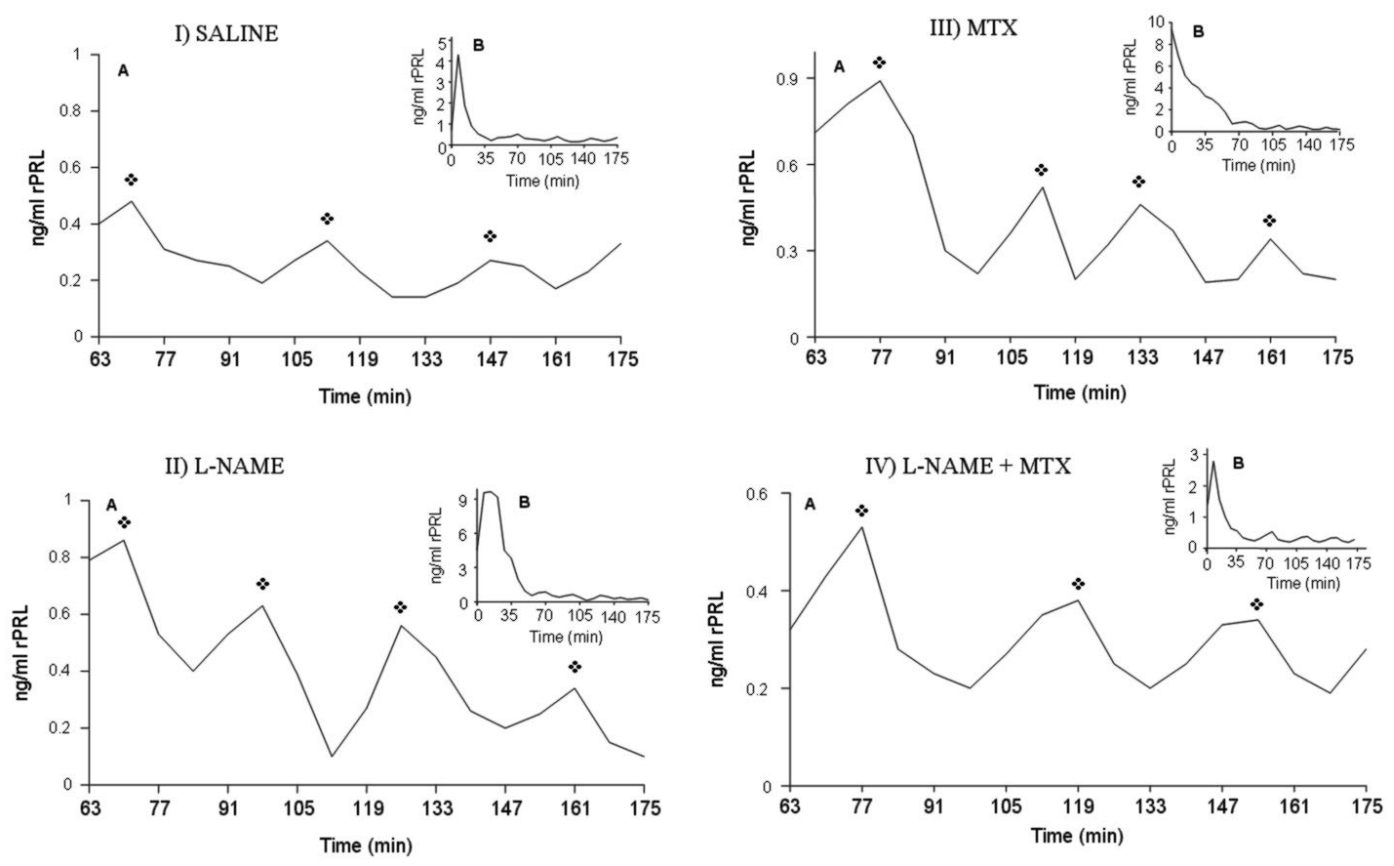
**Individual episodic prolactin patterns during the whole bleeding period (B) and expanded detail of the pulsatile pattern of prolactin during the last 2 hr and 25 min of bleeding (A). **The arrows indicate the prolactin pulses during the studied period. The left upper panel (I) shows the pulsatile pattern of prolactin in control rats treated with saline (vehicle of L-NAME). The left lower panel (II) shows the pulsatile pattern of prolactin in rats treated with L-NAME (rats were ip administered N^ω^-nitro-L-arginine methyl ester at a dose of 10 mg/kg in saline, 60 minutes before the beginning of the bleeding period). The right upper panel (III) shows the pulsatile pattern of prolactin in rats treated with MTX (animals were sc treated with MTX al a dose of 25 mg/kg/day for 1 month). The right lower panel (IV) shows the pulsatile pattern of prolactin in rats treated with MTX and L-NAME (the animals were sc treated with methoxychlor (25 mg/kg/day) for 1 month and L-NAME at a dose of 10 mg/kg in saline, 60 minutes before beginning of the bleeding period).

Prolactin release in animals from the four experimental groups analyzed was episodic, and a representative profile from one animal of each experimental group is shown in Figure 1. As is typical in male rats, the circadian rhythm of prolactin shows a large pulse during the first hours of the morning [31]. Therefore, sample values obtained during the last 2 hr and 25 min of blood withdrawal were used to profile the episodic pattern of prolactin release.

The mean plasma prolactin levels during the bleeding period and the absolute pulse amplitude were increased after MTX administration (Table 1; P ≤ 0.05 and P ≤ 0.001 vs. control group, respectively). In addition, the relative pulse amplitude diminished in MTX-treated rats as compared with the values found in the control group (Table 1; P ≤ 0.01). However, the frequency and duration of prolactin peaks and half-life of the hormone were not modified by the treatment with the pesticide (Table 1). L-NAME increased mean plasma levels of prolactin during the bleeding period and the absolute amplitude of prolactin peaks, as compared to controls (Table 1; P ≤ 0.01 and P ≤ 0.05, respectively). However, L-NAME did not modify any other parameter studied to evaluate prolactin pulsatility (Table 1). L-NAME administration to rats treated with the pesticide reduced mean plasma prolactin levels and the absolute amplitude of prolactin peaks (Table 1). Peak duration, relative amplitude and frequency of prolactin peaks were not change in the group of rats treated with MTX plus L-NAME as compared to either control or MTX treated rats. However, there was an increase in the mean half-life of the hormone and a decrease in the absolute amplitude of prolactin peaks as compared to the MTX treated group (P ≤ 0.05). There was also a reduction in the relative amplitude of the prolactin peaks as compared to the values observed in control rats (Table 1; P ≤ 0.01).

**Table 1:** Mean serum prolactin levels, absolute and relative pulse amplitude, frequency and duration of the pulses, and mean half-life of prolactin, in adult male rats.

**Group**	**rPRL-RP3 (ng/mL)**	**Absolute Amplitude (ng/mL)**	**Relative amplitude (%)**	**Frequency (Pulses/3 h)**	**Duration (min)**	**Half-life (min)**
**Control**	0.56 ± 0.11	0.43 ± 0.05	1.92 ± 0.23	5.17 ± 0.4	29.87 ± 3.92	20.19 ± 2.37
**L-NAME**	0.97 ± 0.10**	0.59 ± 0.03*	1.29 ± 0.39	4.90 ± 0.2	30.03 ± 2.39	19.06 ± 1.44
**MTX**	1.26 ± 0.26*	0.73 ± 0.08**	0.85 ± 0.17**	5.50 ± 0.5	27.01 ± 1.56	16.18 ± 0.81
**MTX + L-NAME**	1.02 ± 0.27	0.32 ± 0.11^#,&^	0.77 ± 0.21**	5.25 ± 0.4	29.50 ± 2.10	25.30 ± 3.74^#^

The relative pulse amplitude was calculated as the quotient between absolute pulse amplitude and preceding nadir value. Values are expressed as mean ± S.E.M. The number of animals per group is 8. *P ≤ 0.05 ≤; **P ≤ 0.01 vs. Control; ^#^P ≤ 0.05 vs. MTX group; ^&^P ≤ 0.05 vs. NAME group.

**Control group: **the animals were ip injected with saline 60 minutes before beginning of the bleeding period, and vehicle 0.5 mL of sesame oil (vehicle in which MTX was administered in MTX group) for 1 month.

**L-NAME group: **the animals were i.p. administered N^w^-nitro-L-arginine methyl ester (L-NAME) at a dose of 10 mg/kg in saline, 60 minutes before beginning of the bleeding period.

**MTX group: **the animals were sc treated with methoxychlor (25 mg/kg/day) for 1 month.

**MTX + L-NAME group: **the animals were s.c. treated with methoxychlor (25 mg/kg/day) for 1 month and L-NAME at a dose of 10 mg/kg in saline, 60 minutes before beginning of the bleeding period.

**Figure 2:**
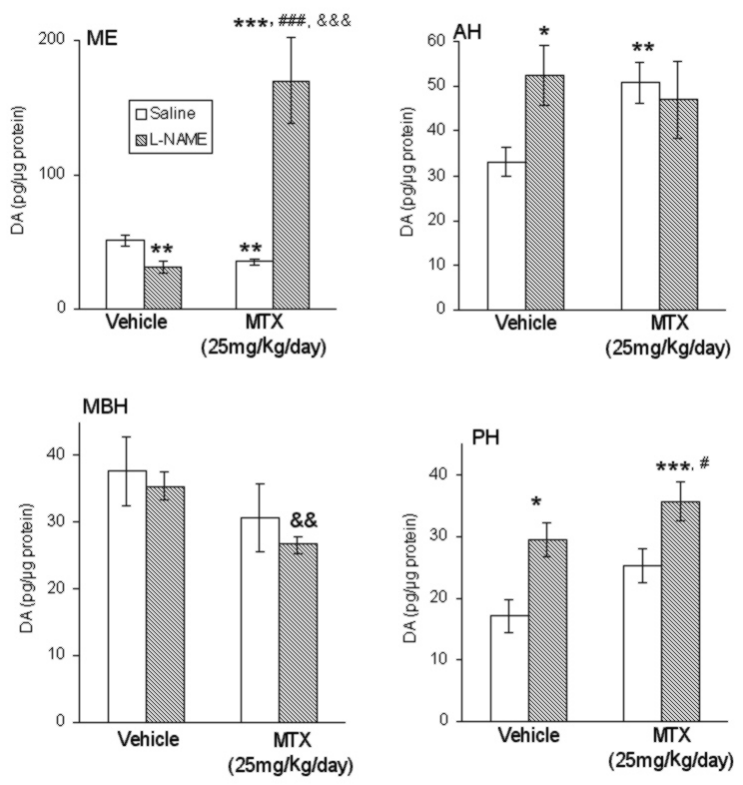
**Dopamine (DA) content in the median eminence (ME), and in the anterior (AH), mediobasal (MBH) and posterior hypothalamus (PH) in the adult control group, MTX-treated rats, L-NAME-treated animals and MTX plus L-NAME-treated rats. **The values are expressed as mean ± S.E.M. (n = 8 in each group). *P ≤ 0.05; **P ≤ 0.01 and ***P ≤ 0.001 vs. control group; ^#^P ≤ 0.05 and ^##^P ≤ 0.01 vs. MTX group; ^&&^P ≤ 0.01 and ^&&&^P ≤ 0.001 vs. L-NAME group.

Whereas MTX decreased DA content in the ME and increased it in the the AH (Figure 2; P ≤ 0.01 vs. control group), its content did not change in the MBH or PH as compared to the values found in the control group (Figure 2).

L-NAME administration decreased DA content in the ME (P ≤ 0.01) as compared to the values found in controls (Figure 2). However, L-NAME administration to MTX exposed rats greatly increased DA content as compared to either MTX treated or control rats (P ≤ 0.001 for any comparison, Figure 2).

In the AH, L-NAME administration increased DA content (P ≤ 0.05) as compared to the values found in non-treated rats, but in MTX-exposed rats this enzymatic inhibitor did not modify DA content as compared to either MTX treated or control rats (Figure 2).

L-NAME administration did not modify DA content at the MBH neither in saline treated nor in MTX treated rats. However, the values of DA in MTX plus L-NAME treated animals were statically decreased as compared to L-NAME treated rats (P ≤ 0.01, Figure 2).

In the PH, L-NAME administration increased DA content (P ≤ 0.05, Figure 2) as compared to the values found in non-treated animals. In addition, in MTX exposed rats this chemical also increased DA content as compared to either MTX treated or control rats (P ≤ 0.05 and P ≤ 0.001 respectively, Figure 2).

There was a statistically significant interaction between MTX and L-NAME on the mean serum prolactin levels (F = 4.46; P ≤ 0.05), absolute pulse amplitude (F = 14.58; P ≤ 0.01), half-life of the hormone (F = 7.85; P ≤ 0.01), and DA concentration in the ME (F = 219.96; P ≤ 0.001).

## Discussion

The results of this study suggest the existence of interaction between L-NAME and MTX to regulate the ultradian secretory pattern of prolactin. The associated changes in the median eminence of DA content may explain the changes in prolactin secretion mediated by MTX and L-NAME.

The observed decline in serum prolactin levels between 10.30 and 11.30 in the control group, may be due to the existence of circadian variations of the hormone previously described [38], and agrees with previous works from our group [31,37,39].

Both MTX and L-NAME administration modified the ultradian secretory pattern of prolactin showing increased mean levels of the hormone during the bleeding period. These results may be explained by the increased absolute amplitude of prolactin peaks found in this study with both MTX and L-NAME treatments. However, after MTX treatment, our data do not agree with the results obtained by Goldman [4], who did not find significant changes in circulating levels of the hormone, although the prolactin content in the pituitary increased. Differences may be due to the length of MTX treatment (2 months in Goldman's work vs. 1 month in our study), dosage approach (oral in Goldman's work vs. subcutaneously in our study), age of the animals at the beginning of pesticide exposure (21 days in Goldman's work vs. adult age in our study), or the season in which the experiment was performed [40]. Also, our present results differ from previous work reported in the literature, where opposite [41] or no effects were observed after L-NAME administration [19,20,33,42] when analyzed at single point assay. The discrepancies may be attributed to the differences in the experimental approaches used in each study: route of administration of L-NAME, dose of L-NAME used, or time of the day in which the experiment was carried out. However, our data obtained in L-NAME-treated animals confirms and extends previous studies describing the inhibitory effect of NO on prolactin secretion, both in vivo and in vitro [43,19,24,16,44,43]. A direct effect of NO at the pituitary level must be considered in light of previous studies [24], although an indirect effect at the hypothalamic or the ME level changing DA synthesis cannot be discarded.

The data obtained after MTX and L-NAME administration indicate the existence of an interaction between the pesticide and NO to modulate prolactin secretion. The increase in AH DA content together with the decrease in this neurotransmitter in the ME suggests that less DA is released from the AH to the ME. These changes may explain the observed modifications in prolactin secretion in this study, as DA is the main inhibitory neuromodulator of prolactin secretion [45,46]. The variations in the AH may be also due to a direct effect of the hormone on the hypothalamus to regulate its own secretion, as has been previously suggested [47]. On the other hand, L-NAME administration induced differential effects on hypothalamic and ME dopamine contents. The interaction between NO and dopamine has been previously demonstrated by Yen and Pan [23]. The changes in DA content in the median eminence, induced by L-NAME, may account for the observed modifications in mean levels of prolactin, considering that the amount of DA released to the pituitary is directly related to its concentration in the ME [48] and that this amine is the main inhibitory input for prolactin secretion [49]. However, other neuroimmunomodulators not monitoredin our study could also be changed by modifications in NO production [19,20,24,33,44].

When MTX and L-NAME were administered together, the mean values of the hormone did not vary compared to the control group. This may be explained by a direct effect on the pituitary inducing the reduction of the absolute amplitude of the prolactin peaks. Also, an indirect effect mediated by the changes observed in ME DA content may be considered.

The changes in DA content observed in the PH indicate that MTX and L-NAME do not interact to change this amine in this hypothalamic area. Interestingly only L-NAME seems to be effective to modify DA in this hypothalamic region, as MTX alone was not able to change the content of this neurotransmitter. These modifications may be associated with changes in autonomic nervous system activity, an effect previously described in other regions of the autonomic nervous system [50].

In summary the results obtained in this work indicate the existence of interactions between MTX and L-NAME at the pituitary level to regulate the ultradian secretory pattern of prolactin. The possibility of an indirect effect mediated by changes in DA content at the ME requires further examination.

## Competing interests

The author(s) declare that they have no competing interest.

## Authors' contributions

AL and AIE designed the experiments. AL and PC carried out the radioimmunoassay for prolactin and the analysis of dopamine by HPLC. AL and TC performed the statistical analysis. TC took care of the experimental animals. AL and AIE supervised the study and drafted the manuscript. All authors read and approved the final manuscript.
